# Prevalence of HPV genotypes and their association with reproductive tract inflammation and pregnancy outcomes among reproductive-age women in Ningbo, China: a retrospective cohort study (2016–2020)

**DOI:** 10.1186/s12879-025-10522-4

**Published:** 2025-02-24

**Authors:** Nana He, Xuejing Song, Qifa Song, Huiqing Ding

**Affiliations:** 1 Medical Research Center, The First Affiliated Hospital of Ningbo University, Ningbo, Zhejiang Province China; 2 Department of Obstetrics and Gynecology, The First Affiliated Hospital of Ningbo University, 59 Liuting Road, Ningbo, Zhejiang Province 315000 China

**Keywords:** Human papillomavirus, Reproductive tract inflammation, Adverse pregnancy outcomes, Reproductive-age women, China

## Abstract

**Background:**

The comprehensive impact of human papillomavirus (HPV) infection on reproductive tract inflammation and adverse pregnancy outcomes among women of reproductive age has not been fully investigated.

**Methods:**

A retrospective cohort study was conducted among women presenting to the gynecological departments of specialized women’s hospitals in Ningbo, China, between 2016 and 2020. A total of 6506 women, with an average age of 28.7 years, who had undergone HPV testing and genotyping were included in the study.

**Results:**

Overall, the most prevalent HPV types were HPV 52 (25.8%), HPV 16 (17.9%), and HPV 58 (7.7%). Vaginitis and cervicitis were significantly more prevalent in women with high-risk HPV (HR-HPV) compared to those with low-risk HPV(LR-HPV) (9.0% vs. 4.7%; 10.8% vs.7.1%; *P* < 0.05). Moreover, the incidence of cervicitis in patients with persistent HPV infection for more than two years was significantly higher than in those with HPV infection for one year (21.8% vs. 11.8%; *P* < 0.05). Pregnant women with HR-HPV infection had a significantly increased risk of miscarriage (9.7% vs. 6.1%; *P* < 0.05). Our cross-sectional analysis of potential risk factors for HPV infection during pregnancy revealed that higher pregnancy glucose levels (prevalence, 4.23%; OR, 1.10; 95% CI, 1.10–1.20; *P* < 0.05) significantly increased the risk of HPV infection. Women with persistent HR-HPV infection had a significantly higher risk of reproductive tract inflammation and adverse pregnancy outcomes. The analysis revealed significant associations between HPV infection and several pregnancy outcomes, including an increased risk of miscarriage, reduced live birth rate, and a higher cesarean section rate.

**Conclusions:**

This highlights the need to monitor gestational glucose levels, reproductive tract inflammation, and HPV infection to reduce the risk of adverse pregnancy outcomes among pregnant women.

## Background

Human papillomavirus (HPV) is a virus characterized by its circular double-stranded DNA structure. HPV is sexually transmitted and primarily infects the epidermis and mucous membranes, causing asymptomatic and self-limited infections [1]. High-risk HPV (HR-HPV) genotypes, including HPV 16, 18, 31, 33, 35, 39, 45, 51, 52, 56, 58, 59, 66, and 68, are associated with an increased risk of cervical cancer [1, 2]. Over 99% of cervical cancer cases can be attributed to HR-HPV infections, particularly HPV 16 and 18 [3, 4]. Concurrent HPV infection and reproductive tract inflammation can increase the risk of developing cervical cancer over time [5, 6]. HPV proteins are directly and indirectly involved in the development of chronic inflammation in the reproductive tract, which is a causal factor in the development of cervical cancer [7, 8]. Most current cross-sectional studies indicate that persistent HR-HPV infection can cause dysbiosis of the vaginal microbiota in women, which can cause a local immune inflammatory response and increase the incidence of reproductive tract inflammation [9, 10]. Due to their increasing prevalence, HPV infection and reproductive tract inflammation have become significant threats to women’s reproductive health. Preventing HPV infection in women of reproductive age remains a crucial challenge in clinical practice [11, 12].

Women of reproductive age are at high risk of HPV infection, with a global prevalence of up to 30% in pregnant women, particularly in the meta-late phases of pregnancy [13]. There are several risk factors that influence HPV infection during pregnancy, including age, hormone levels, pregnancy complications, and autoimmunity. Elevated estrogen levels and other hormonal changes in pregnant women can lead to active HPV replication and an increased risk of HPV infection [14, 15]. The prevalence of HPV infection in pregnant women decreases with age, suggesting that age is an important factor for HPV infection [16]. Studies have shown that pregnant women with gestational diabetes are more likely to be infected with HPV [17]. Additionally, HR-HPV infection can increase the risk of reproductive tract inflammation [18, 19] and adverse pregnancy outcomes, including miscarriage, preterm birth, premature rupture of membranes (PROM), and fetal growth restriction [20, 21]. Although there is controversy about whether HR-HPV infection leads to adverse pregnancy outcomes, few studies have investigated the effects of HPV infection on maternal and infant outcomes.

We conducted a real-world retrospective cohort study to investigate the impacts of HPV infection on reproductive tract inflammation and adverse pregnancy outcomes among women of reproductive age. Possible risk factors for HPV infection in pregnant women were analyzed, including age and glucose, estradiol, and progesterone levels. The aim of our study was to provide a scientific basis for the prevention of HPV infection in women of reproductive age.

## Methods

### Study population

The study data were obtained from the electronic health records (EHRs) of 6506 patients who underwent obstetric examinations and HPV testing at the gynecological departments of hospitals in Ningbo, China, between 2016 and 2020. The inclusion criteria were as follows: (1) women aged 18 years or older; (2) nonpregnant women with normal menstruation for more than 3 days; (3) women who did not perform vaginal douching or take medication; (4) women who were willing to provide an adequate cervical specimen for HPV testing and genotyping. The exclusion criteria included (1) malignant tumors or autoimmune diseases and (2) a history of cognitive impairment or psychiatric disorders.

### Follow-up details

#### Start of follow-up

The start of follow-up was uniformly defined as the date of the first HPV test recorded during the study period (2016–2020) for all women, regardless of their HPV status. This ensures that the follow-up period is consistent across all participants, whether they tested positive or negative for HPV during the study.

#### Defining HPV types

For women who had multiple HPV tests at various time points, the HPV type for analysis was determined based on the first positive test result for those who tested positive. If a woman had multiple positive tests, the HPV type from the earliest positive test was used. For women with only negative tests, their HPV status was recorded as negative throughout the study.

This revised approach ensures that each woman’s follow-up period is clearly defined from the initial test date, and the HPV type is consistently categorized based on the first positive result.

### Ethical statement

This study used EHR data, which was kept confidential throughout the study in accordance with all ethical and professional standards; therefore, ethical approval was waived by the Ethics Committee of Ningbo First Hospital, and all research was performed in accordance with the Declaration of Helsinki.

### Collection of clinical feature data

Data on maternal clinical features, including sex, age, diagnosis, and symptoms noted during routine obstetric examinations; descriptive statistics, including the mode of delivery (the frequency of each type of vaginal and/ or cesarean, etc.) and pregnancy outcomes (the number of preterm births, puerperal infection, postpartum hemorrhage, and PROM); and past medical history (diabetes, hypertension, and thyroid disease) were collected. The neonatal data included a 1-minute Apgar score and a 5-minute Apgar score for the neonatal assessment. These scores consisted of the heart rate, breathing rate, response to stimulation, muscle tone, and skin color, with a score range of 0–2 for each item and a total score of 10 points. The diagnostic criteria are 8–10 for normal, 4–7 for mild asphyxia, and 0–3 for severe asphyxia. The 1-minute score can distinguish the degree of asphyxia, while the 5-minute score can help determine the resuscitation effect and prognosis.

### Laboratory measurements

Standard laboratory measurements included the hematocrit (HCT) level, white blood cell (WBC) count, total protein (TP) level, globulin (GLB) level, human serum albumin (HSA) level, C-reactive protein (CRP) level, glycated hemoglobin, type hemoglobin A1C (HbA1c) level, and premature rupture of membranes (PROM) status. Overnight fasting blood was drawn from participants. Triglyceride (TG), total cholesterol (TC), low-density lipoprotein cholesterol (LDL-C), high-density lipoprotein cholesterol (HDL-C), and lipoprotein (a) (Lp (a)) levels were determined using standard immunological and enzyme procedures (IFCC recommendations) on the Beckman Coulter AU5800 analyzer (Beckman Coulter Inc., Brea CA, USA). HbA1c was measured using high-performance liquid chromatography. Biochemical tests, including total protein, globulin, serum albumin, estradiol levels, and progesterone levels, were performed during the second trimester of pregnancy, specifically between 20 and 24 weeks of gestation. All biochemical assays were assessed by the Interlaboratory Quality Assessment Program of the Chinese Ministry of Health to ensure reliable results.

### Obstetric examinations

Patients underwent prenatal and obstetric examinations, including clinical cytological and microbiological examinations. Microbiological examinations, specifically including the vaginal microecology test, were conducted to assess the presence and balance of microorganisms in the vaginal environment. A physical examination of the genital area was performed to inspect the cervix and vagina for signs of inflammation. HPV testing was conducted at the initial gynecological examination and repeated as needed based on clinical guidelines and patient history. Laboratory tests and clinical information were collected at multiple time points, including the initial examination, follow-up visits, and during pregnancy and delivery if applicable. For detailed procedures and diagnostic criteria, please refer to the appendix.

### HPV testing and genotyping

The cervical surface was scraped by cervical brushes after the cervix uteri had been completely exposed with a vaginal dilator, inserted 1–1.5 cm into the endocervical canal. The cervical cell samples were used to test for the HPV at the hospital’s clinical laboratory. These samples were collected using a cytobrush during the gynecological examination. A polymerase chain reaction-reverse dot blot (PCR-RDB) assay was used to identify 23 genotypes, including 14 h-HPV genotypes (HPV 16, 18, 31, 33, 35, 39, 45, 51, 52, 56, 58, 59, and 68) [22, 23, 24, 25], four possible HR-HPV genotypes (53, 66,73, 82, and 83) and LR-HPV genotypes (HPV 6, 11, 42, 43, and 81). The study included HPV results that were obtained by PCR-RDB in laboratories that participated in the national interlaboratory quality evaluation.

### Statistical analysis

**Fig. 1:**
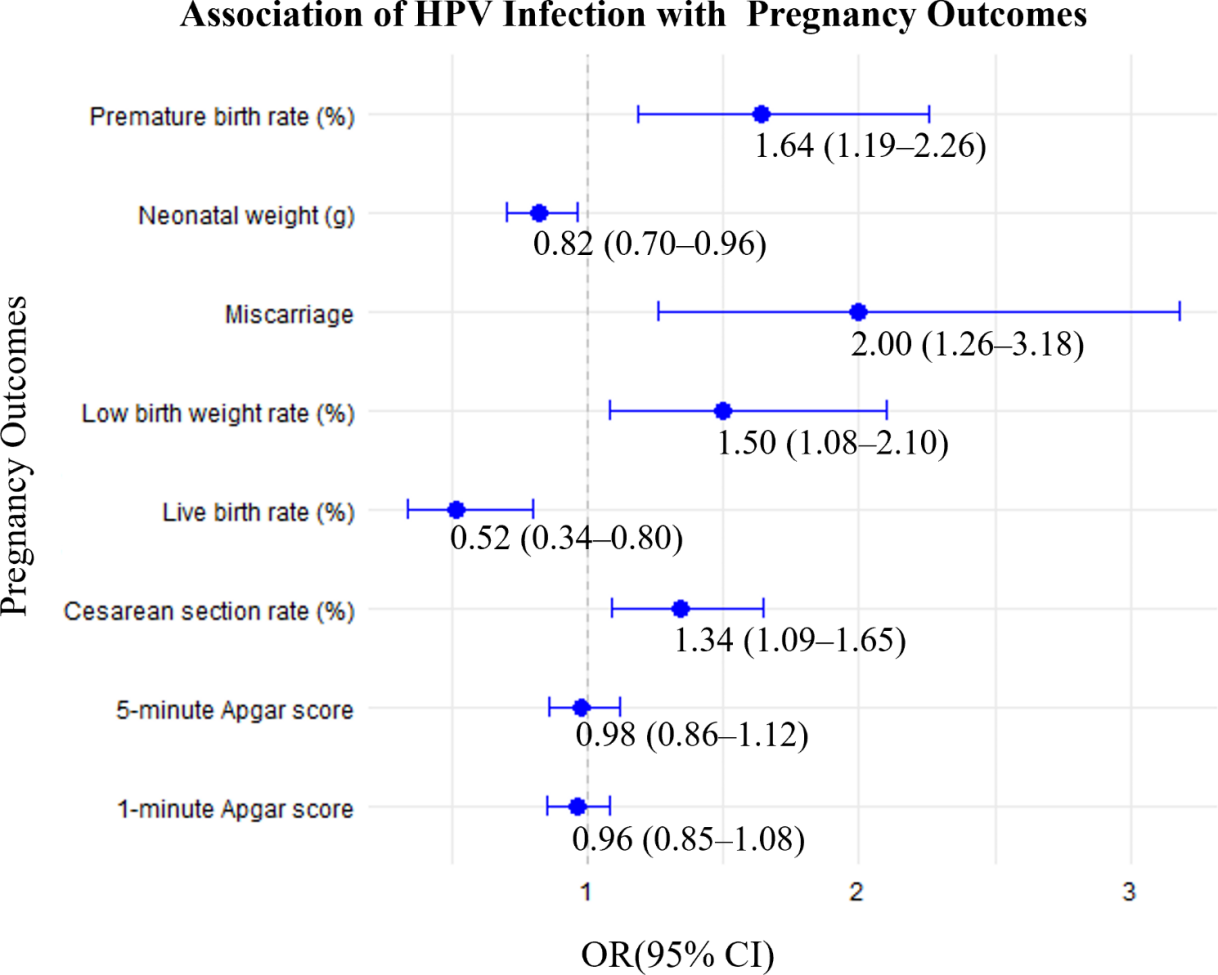
Odds ratios (ORs) for HR-HPV infection associated with potential risk of adverse pregnancy outcomes

**Fig. 2:**
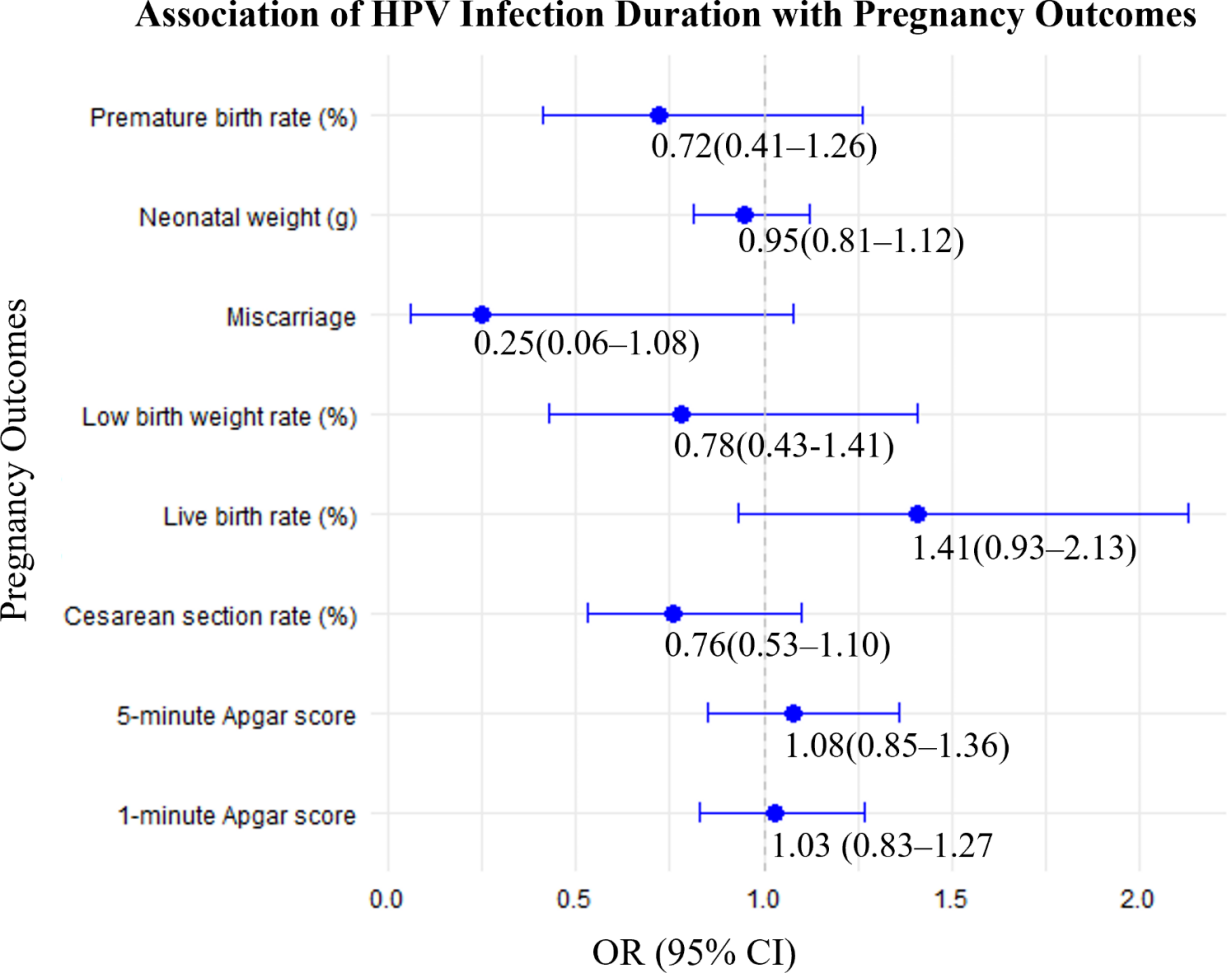
Odds ratios (ORs) for HPV infection duration associated with potential risk of adverse pregnancy outcomes

HPV infection levels are expressed as the incidence with a unit of /1000 person-year. Normally distributed continuous variables are expressed as mean (standard deviation [SD]). The means of two continuous variables were compared using independent samples and Student’s t-tests. Categorical variables were presented as numbers (%). For comparisons between groups, the chi-square test was used when the expected frequency in each cell was 5 or greater. In cases where the expected frequency in any cell was less than 5, Fisher’s exact test was employed to ensure accuracy. Statistical significance was determined by *P* values, with a threshold of *P* < 0.05 considered significant. Then, we compared the prevalence odds ratio (OR) of HPV infection by age and glucose, estradiol, and progesterone levels. Two-tailed *p*-values less than 0.05 were considered statistically significant. The statistical analyses for Figs. 1 and 2 employed logistic regression models to assess the impact of HPV infection and its duration on various pregnancy outcomes. For Fig. 1, the analysis compared high-risk HPV (HR-HPV) with low-risk HPV (LR-HPV) using crude odds ratios (ORs) without adjusting for confounders, with LR-HPV serving as the reference group. For Fig. 2, the effect of HPV infection duration (more than two years vs. one year) was evaluated using crude ORs, with the one-year HPV infection group as the reference. These models provide insights into the associations of HPV infection and its duration with pregnancy outcomes.

## Results

### Prevalence of different HPV genotypes

A total of 6506 women of reproductive age were included in this study, and all of them underwent HPV testing. This study included 1069 HPV-positive and 5437 HPV-negative women (mean age, 28.6 ± 4.62 and 28.9 ± 4.60 years, respectively). The overall rate of any HPV among women who underwent HPV testing was 16.4% (1069/6506). A total of 13,752 HPV tests were conducted on 6,506 subjects from 2016 to 2021, of which 1,751 tested positive for HPV and 133,001 tested negative. In addition, the number of HPV testing per woman ranged from 1 to 3. A total of 29 HPV genotypes (17 h-HPV, two possible HR-HPV and ten LR-HPV genotypes) were detected (Table 1). Women in the HPV-positive group, had a single HPV type with 84.8% (1485/1751) testing positive for an HR-HPV or a putative HR-HPV genotype, and 15.2% (266/1751) testing positive for an LR-HPV genotype. In HPV-infected women, the three most prevalent genotypes, HPV 52, HPV 16, and HPV 58, accounted for 25.8% (453/1751), 17.9% (313/1751), and 7.7% (135/1751) of all positive cases (including coinfection), respectively. HPV 81 (3.1%, 55/1751), HPV 6 (3.0%, 52/1751) and HPV 11 (2.8%, 49/1751) were the three most prevalent LR-HPV types.

**Table 1:** Distribution of individual HPV genotypes

HPV genotypes	No. of positive tests	Over all incidence among 6506 women with total incidence time 20,762 person-years (/1000 person-years)	Composition proportion (%)
**HR-HPV (Total number of events: 1469)**			
HPV-52	453	21.8	25.8%
HPV-16	313	15.1	17.9%
HPV-58	135	6.5	7.7%
HPV-51	78	3.8	4.5%
HPV-53	73	3.5	4.2%
HPV-56	63	3.0	3.6%
HPV-18	63	3.0	3.6%
HPV-39	52	2.5	3.0%
HPV-68	45	2.2	2.6%
HPV-66	41	2.0	2.3%
HPV-59	40	1.9	2.3%
HPV-33	37	1.8	2.1%
HPV-31	27	1.3	1.5%
HPV-45	23	1.1	1.3%
HPV-35	13	0.6	0.74%
HPV-82	9	0.4	0.51%
HPV-73	4	0.2	0.23%
**Putative HR-HPV (Total number of events: 16)**			
HPV-53	12	0.5	0.69%
HPV-26	4	0.2	0.23%
**LR-HPV (Total number of events: 266)**			
HPV-81	55	2.6	3.1%
HPV-6	52	2.5	3.0%
HPV-11	49	2.4	2.8%
HPV-42	30	1.4	1.7%
HPV-43	28	1.3	1.6%
HPV-44	13	0.6	0.74%
HPV-54	12	0.6	0.69%
HPV-40	11	0.5	0.63%
HPV-61	10	0.5	0.57%
HPV-2	6	0.3	0.34%

Note: HR-HPV: high-risk HPV; LR-HPV: low-risk HPV

### Comparison of clinical features between HPV-positive and HPV-negative patients

As the number of HPV testing per woman ranged from 1 to 3 during the study, the total incidence follow-up time was 3191 person-years for 1069 women with HPV and 19,733 person-years for 5437 women without HPV (Table 2). The two groups were comparable in terms of age. Regarding the laboratory measures, the HPV-positive group showed significantly higher serum albumin (42.97 ± 4.14 g/L vs. 41.35 ± 4.34 g/L, *P* < 0.001) levels and significantly lower Hct (37.61 ± 4.53% vs. 38.71 ± 3.88%, *P* < 0.001), WBC (7.25 ± 2.4810^9^/L vs. 7.81 ± 2.5510^9^/L, *P* < 0.001), CRP (8.71 ± 13.73 mg/L vs. 11.94 ± 16.02 mg/L, *P* < 0.001), HbAlc (5.13 ± 1.71% vs. 5.40 ± 1.03%, *P* < 0.001), glucose (5.13 ± 0.96 mmol/L vs. 5.15 ± 0.87 mmol/L, *P* < 0.001), progesterone (29.89 ± 32.28 nmol/L vs. 37.21 ± 32.4 nmol/L, *P* < 0.001) and estradiol levels (1104 ± 1633 pmol/L vs. 1539 ± 2077 pmol/L, *P* < 0.001) than the HPV-negative group. In addition, we explored the impact of HPV infection on reproductive tract inflammation. The HPV-positive group showed an increased incidence of vaginitis, cervicitis and mycoplasma infections, as compared to HPV-negative group (*P* < 0.01). There were no statistically significant differences in the incidence of pelvic inflammation, acute urethritis, or endometritis between the two groups. Finally, we explored the impact of HPV infection on pregnancy and neonatal outcomes. HPV-positive patients had a significantly higher incidence of miscarriage and PROM than HPV-negative patients (*P* < 0.01). However, there were no significant differences in neonatal weight and Apgar scores between the two groups.

**Table 2:** Comparison of clinical features between HPV-positive and HPV-negative women

	HPV (-) (19733 person-years)	HPV (+) (3191 person-years)		*p* value
Age (year)	28.9 ± 4.60	28.6 ± 4.62	< 0.001^**^	
Hct (%)	38.71 ± 3.88	37.61 ± 4.53	< 0.001^**^	
WBC (10^9^/L)	7.81 ± 2.55	7.25 ± 2.48		< 0.001^**^
TP (g/L)	69.57 ± 5.48	71.46 ± 5.2		0.1328
Alb (g/L)	41.35 ± 4.34	42.97 ± 4.14		< 0.001^**^
CRP (mg/L)	11.94 ± 16.02	8.71 ± 13.73		< 0.001^**^
HbAlc (%)	5.4 ± 1.03	5.13 ± 0.71		< 0.001^**^
Glucose (mmol/L)	5.15 ± 0.87	5.13 ± 0.96		< 0.001^**^
Progesterone (nmol/L)	37.21 ± 32.4	29.89 ± 32.28		< 0.001^**^
Estradiol (pmol/L)	1539 ± 2077	1104 ± 1633		< 0.001^**^
**Reproductive tract illness (n**,** /1000 person-years)**				
Vaginitis	1270 (64)	347 (109)		< 0.001^**^
Cervicitis	432 (22)	133 (42)		< 0.001^**^
Pelvic inflammation	205 (10)	27 (8)		0.366
Mycoplasma infections	69 (3)	29 (9)		< 0.001^**^
Acute urethritis	124 (6)	29 (9)		0.093
Endometritis	45 (2)	15 (5)		0.022^*^
**Pregnancy outcome**				
Miscarriage (n, /1000 person-years)	1447 (97)	363 (114)		0.00922^**^
PROM (n, /1000 person-years)	1406 (94)	296 (93)		0.00319^**^
Neonatal weight (g)	3254 ± 544.2	3268 ± 553.6		0.448
1-minute Apgar score	9.35 ± 1.13	9.35 ± 1.22		0.976
5-minute Apgar score	9.86 ± 1.04	9.83 ± 1.12		0.338

Note: Continuous data are expressed as mean ± SD. Enumerating data are expressed as number divided by total incidence time (/1000 person-years)

Hct: hematocrit; WBC: white blood cell; TP: total protein; Glo: globulin; Alb: albumin; CRP: C-reactive protein; HbAlc: glycated hemoglobin; PROM: premature rupture of membranes

HR-HPV and LR-HPV infections have different distributions in women of reproductive age, which may result in varying degrees of genital tract inflammation and adverse pregnancy outcomes. The baseline characteristics of the HR-HPV-positive and LR-HPV-positive populations are shown in Table 3. In analyzing biochemical parameters, we observed that serum albumin (41.13 ± 4.47 g/L vs. 42.03 ± 4.68 g/L, *p* < 0.05), CRP (1.98 ± 2.16 mg/L vs. 7.06 ± 11.50 mg/L, *p* < 0.05) levels, and HbAlc (5.20 ± 0.92 mmol/L vs. 5.45 ± 1.08 mmol/L, *P* < 0.01) levels were significantly lower in HR-HPV-positive subjects than in LR-HPV-positive subjects (*P* < 0.05). There were no statistically significant differences between the HR-HPV-positive group and LR-HPV-positive group in terms of other indicators. Then, we examined the effects of HR-HPV and LR-HPV infections on inflammation of the reproductive tract. There was a significantly increased incidence of vaginitis and cervicitis in the HR-HPV positive group compared with the LR-HPV positive group (*P* < 0.05). The incidence of pelvic inflammation, mycoplasma infections, acute urethritis, and endometritis was not statistically different between the two groups. Finally, we examined the impact of HR-HPV and LR-HPV infections on adverse pregnancy outcomes and neonatal outcomes. Our data showed that the incidence of miscarriage in HR-HPV-positive women was significantly higher than that in LR-HPV-positive women (*P* < 0.05). We also observed that the neonatal weight (3247 ± 566 g vs. 3388 ± 587 g, *P* < 0.01) was significantly lower in the HR-HPV-positive group than that in the LR-HPV-positive group (*P* < 0.05). There were no significant differences in Apgar scores between the two groups.

**Table 3:** Comparison of clinical features between HR-HPV-positive and LR-HPV-positive women

	HR-HPV (+) (2768 person-years)	LR-HPV (+) (423 person-years)	OR (95% CI)	*p* value
Age (years)	29.1 ± 4.7	28.9 ± 4.6	NA	0.674
Hct (%)	38.01 ± 5.92	37.51 ± 6.78	NA	0.223
WBC (10^9^/L)	7.52 ± 3.55	7.63 ± 2.92	NA	0.613
TP (g/L)	70.41 ± 5.9	69.83 ± 5.91	NA	0.239
Glo (g/L)	28.25 ± 3.38	28.13 ± 3.48	NA	0.682
Alb (g/L)	42.03 ± 4.68	41.13 ± 4.47	NA	0.029^*^
CRP (mg/L)	7.06 ± 11.50	1.98 ± 2.16	NA	0.042^*^
HbAlc (mmol/L)	5.45 ± 1.08	5.20 ± 0.92	NA	< 0.001
Glucose (mmol/L)	5.29 ± 1.12	5.33 ± 1.6	NA	0.749
Progesterone (nmol/L)	28.06 ± 34.3	34.32 ± 34.15	NA	0.094
Estradiol (pmol/L)	1106 ± 2001	1578 ± 2353	NA	0.057
**Reproductive tract illness (n**,** /1000 person-years)**				
Vaginitis	249 (90)	20 (47)	NA	0.003^**^
Cervicitis	299(108)	30 (71)	NA	0.019^*^
Pelvic inflammation	28 (10)	3 (7)	NA	0.555
Mycoplasma Infections	27 (10)	1 (2)	NA	0.129
Acute urethritis	50 (18)	11(26)	NA	0.266
Endometritis	9 (3)	1 (2)	NA	0.761
**Pregnancy outcomes**				
Miscarriage (n, /1000 person-years)	97 (97)	26 (61)	2.00 (1.26–3.18)	0.009^**^
Neonatal weight (g)	3247 ± 566	3388 ± 587	0.82 (0.70–0.96)	< 0.001^**^
1-minute Apgar score	9.29 ± 1.4	9.32 ± 1.43	0.96 (0.85–1.08)	0.735
5-minute Apgar score	9.77 ± 1.34	9.79 ± 1.35	0.98 (0.86–1.12)	0.767
Live birth rate (%)	88.5	93.0	0.52 (0.34–0.80)	0.002^**^
Cesarean section rate (%)	45.2	37.8	1.34 (1.09–1.65)	0.015^*^
Premature birth rate (%)	12.5	8.0	1.64 (1.19–2.26)	0.020^*^
Low birth weight rate (%)	9.0	6.5	1.50 (1.08–2.10)	0.030^*^

Note: Continuous data are expressed as mean ± SD. Enumerating data are expressed as number divided by total incidence time (/1000 person-years)

Hct: hematocrit; WBC: white blood cell; TP: total protein; Glo: globulin; Alb: albumin; CRP: C-reactive protein; HbAlc: glycated hemoglobin; PROM: premature rupture of membranes

Persistent HPV infection is associated with an increased risk of developing reproductive tract inflammation and adverse pregnancy outcomes in women. Thus, we further analyzed the impact of HPV infection on reproductive tract inflammation and adverse pregnancy outcomes under different infection durations. The HPV-positive patients were divided into two groups according to the duration of infection, with 765 person-years in the group with an HPV infection duration of one year and 147 person-years in the group with an HPV infection duration of more than two years (Table 4). The total protein (70.42 ± 5.19 g/L vs. 71.79 ± 5.23 g/L, *p* < 0.05), globulin (27.68 ± 2.90 g/L vs. 28.41 ± 3.22 g/L, *p* < 0.05), and serum albumin (42.17 ± 4.21 g/L vs. 43.21 ± 4.12 g/L, *p* < 0.05) levels in patients with an HPV infection duration of two years were significantly lower than in patients with an HPV infection duration of one year (*P* < 0.05). Notably, the progesterone level (38.13 ± 36.92 nmol/L vs. 27.98 ± 31.19 nmol/L, *P* < 0.05) in patients with an HPV infection duration of two years was significantly higher than that in patients with an HPV infection duration of one year. At the same time, the incidence of cervicitis in patients with an HPV infection duration of more than two years was significantly higher than that in patients with an HPV infection duration of one year (*P* < 0.05). However, there were no significant differences in adverse pregnancy and neonatal outcomes between the two groups.

**Table 4:** Comparison of clinical features between one-year and over one-year infection patients

	HPV-positive for 1 year (765 person-years)	HPV-positive for over 2 years (147 person-years)	OR (95% CI)	*p* value
Age (years)	28.9 ± 4.60	28.6 ± 4.62	NA	< 0.001^**^
Hct (L/L)	38.45 ± 5.31	38.07 ± 4.47	NA	0.4644
WBC (10^9^/L)	7.29 ± 2.57	6.98 ± 2.30	NA	0.2014
TP(g/L)	71.79 ± 5.23	70.42 ± 5.19	NA	0.0255^*^
Glo(g/L)	28.41 ± 3.22	27.68 ± 2.90	NA	0.0498^*^
Alb(g/L)	43.21 ± 4.12	42.17 ± 4.21	NA	0.0461^*^
CRP (mg/L)	8.32 ± 12.91	9.81 ± 15.13	NA	0.3783
HbAlc (mmol/L)	5.74 ± 1.01	6.44 ± 1.69	NA	0.1706
Glucose (mmol/L)	5.31 ± 0.96	5.39 ± 0.97	NA	0.5202
Progesterone (nmol/L)	27.98 ± 31.19	38.13 ± 36.92	NA	0.0408^*^
Estradiol (pmol/L)	1124 ± 1725	1185 ± 1458	NA	0.8394
**Reproductive tract diseases (%)**				
Vaginitis (n, %)	90 (11.8%)	16 10.8%)	NA	0.760
Cervicitis (n, %)	90(11.8%)	32(21.8%)	NA	0.0012^**^
Pelvic inflammation (n, %)	6(0.8%)	1(0.7%)	NA	0.4133
Mycoplasma infections (n, %)	7(0.9%)	0(0)	NA	NA
Acute urethritis (n, %)	5(0.7%)	2(1.4%)	NA	0.7013
Endometritis (n, %)	4(0.5%)	1(0.7%)	NA	0.1871
**Adverse pregnancy outcome (%)**				
Miscarriage (n, %)	5(0.7%)	4(2.7%)	0.25(0.06–1.08)	0.0619
neonatal weight (g)	3221 ± 572.5	3281 ± 545.5	0.95(0.81–1.12)	0.2476
1-minute Apgar score	9.37 ± 1.01	9.34 ± 1.28	1.03 (0.83–1.27	0.8128
5-minute Apgar score	9.89 ± 0.87	9.81 ± 1.18	1.08(0.85–1.36)	0.4577
live birth rate (%)	89.0	85.0	1.41(0.93–2.13)	0.020^*^
Cesarean section rate (%)	43.5	50.3	0.76(0.53–1.10)	0.010^*^
Premature birth rate (%)	13.0	17.5	0.72(0.41–1.26)	0.045^*^
Low birth weight rate (%)	10.0	12.5	0.78(0.43–1.41)	0.038^*^

Note: Continuous data are expressed as mean ± SD. Enumerating data are expressed as number divided by total incidence time (/1000 person-years)

Hct: hematocrit; WBC: white blood cell; TP: total protein; Glo: globulin; Alb: albumin; CRP: C-reactive protein; HbAlc: glycated hemoglobin; PROM: premature rupture of membranes

### Potential increasing risks for HPV infection

**Fig. 3:**
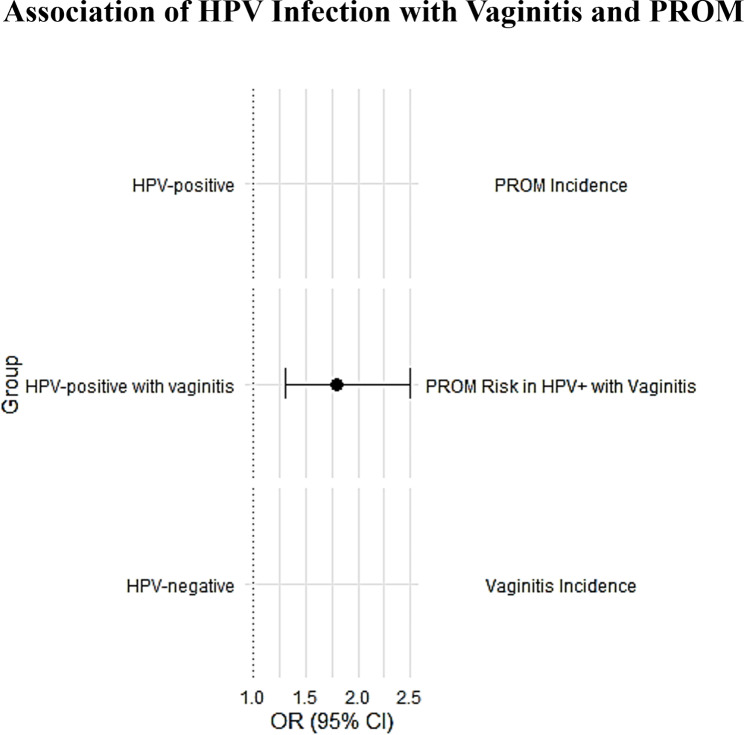
Odds ratios (ORs) for HPV infection associated with potential risk factors in pregnant women

Regarding the results of our cross-sectional analysis of associations between potential risk factors and HPV infection during pregnancy, we investigated the associations in terms of the OR of the prevalence of HPV infection by age and glucose, estradiol, and progesterone levels (Fig. 3). The crude data indicated that the glucose level (GDM) (*N* = 871; prevalence, 4.23%; OR, 1.10; 95% CI, 1.10–1.20; *p* < 0.05) was associated with an increased risk of HPV infection. In contrast, negative associations with HPV infection were found for an age of 25–35 years (*N* = 2313; prevalence, 3.93%; OR, 0.70; 95% CI, 0.61–0.81; *p* < 0.001), an age > 35 years (*N* = 640; prevalence, 3.46%; OR, 0.62; 95% CI, 0.53–0.72; *p* < 0.001), estradiol levels (> 1100 pmol/L) (*N* = 163; prevalence, 3.61%; OR, 0.72; 95% CI, 0.58–0.88; *p* < 0.01), and progesterone levels with two thresholds (2.8–50 nmol/L, > 50 nmol/L) with ORs of 0.76 (95% CI, 0.64–0.90; *p* < 0.01) and 0.64 (95% CI, 0.53–0.77; *p* < 0.001), respectively.

### Association of HPV infection with vaginitis and PROM

**Fig. 4:**
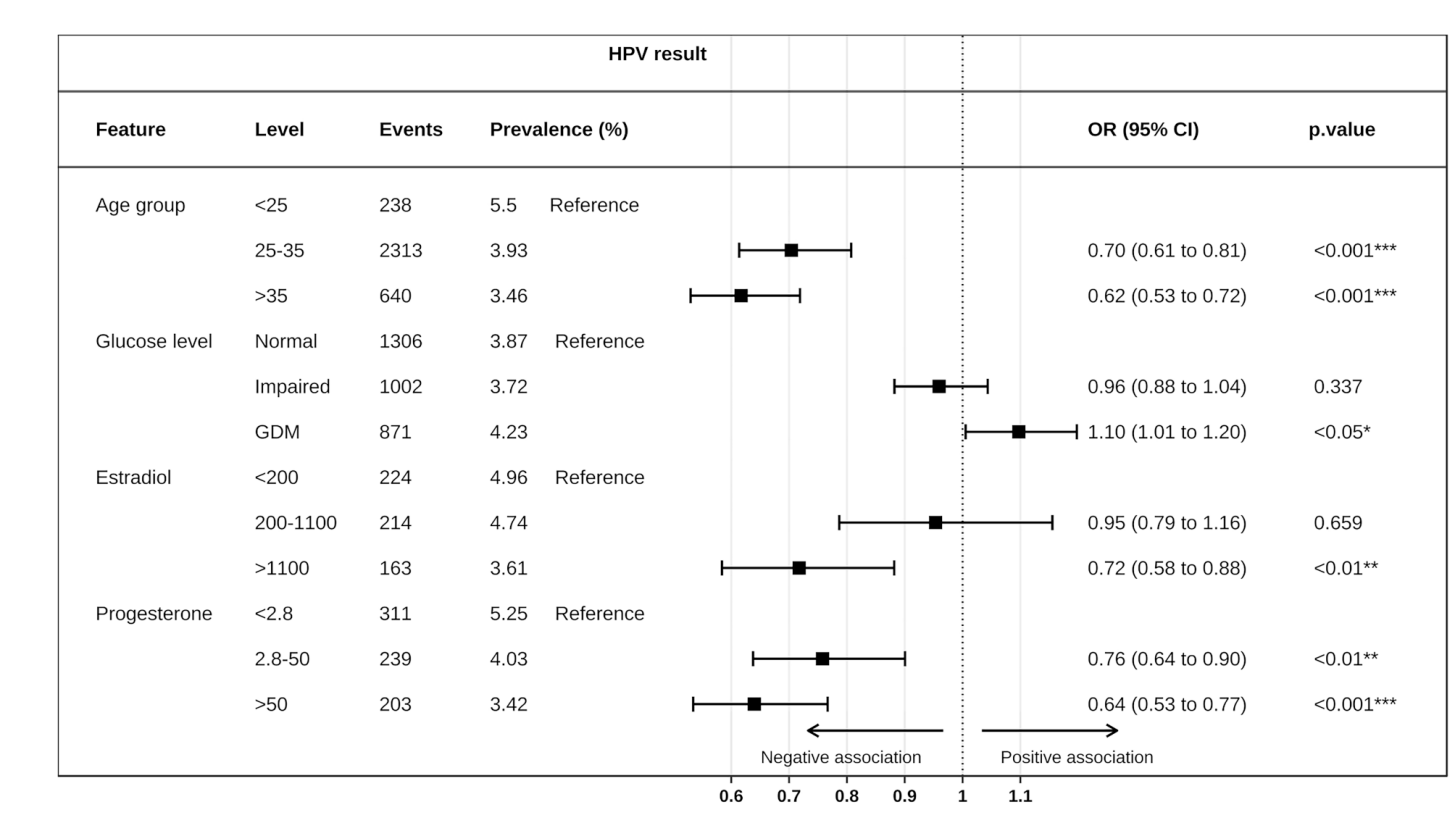
Odds ratios (ORs) for HPV Infection with Vaginitis associated with potential risk of PROM

It is well known that bacterial vaginosis increases adverse pregnancy outcomes. Therefore, we further analyzed whether HPV infection would lead to an increased incidence of PROM due to reproductive tract infection. We have conducted a multivariate logistic regression analysis to assess the association between HPV infection, vaginitis, and the risk of premature rupture of membranes (PROM). The analysis adjusted for potential confounding factors such as age, parity, and gestational age at the time of testing. The results indicated that HPV-positive women with concurrent vaginitis had a significantly higher risk of PROM compared to HPV-negative women. The odds ratio (OR) for PROM in HPV-positive women with vaginitis was 1.8 (95% CI: 1.3–2.5), suggesting a strong association between reproductive tract infection and increased risk of PROM (Fig. 4).

### Association of HR-HPV infection with pregnancy outcomes

To elucidate the potential impact of HR-HPV infection on pregnancy outcomes, we estimated the odds ratios (ORs) for each pregnancy outcome in Table 3. It was demonstrated the association between HPV infection and pregnancy outcomes. HR-HPV infection is significantly associated with an increased risk of miscarriage (OR, 2.00; 95% CI, 1.26–3.18), a lower live birth rate (OR, 0.52; 95% CI, 0.34–0.80), and a higher cesarean section rate (OR, 1.34; 95% CI, 1.09–1.65). Additionally, HR-HPV infection is linked to a higher risk of premature birth (OR, 1.64; 95% CI, 1.19–2.26) and low birth weight (OR, 1.50; 95% CI, 1.08–2.10). However, the impact of HR-HPV on neonatal weight and Apgar scores at 1 and 5 min is less pronounced (Fig. 1).

### Association of HPV infection duration with pregnancy outcomes

To determine the potential impact of duration of HPV infection on pregnancy outcomes, we estimated the odds ratios (ORs) for each pregnancy outcome in Table 4. It was illustrated the effect of HPV infection duration on pregnancy outcomes. Prolonged HPV infection shows some association with a lower risk of miscarriage and cesarean section, but these findings are not statistically significant for most outcomes. The duration of HPV infection does not show a substantial impact on neonatal weight or Apgar scores. While there are trends indicating potential associations with live birth rate, premature birth, and low birth weight, these are not statistically significant. There were no statistically significant differences in the OR values in this graph, suggesting that the duration of infection may not have a statistically significant effect on these pregnancy outcomes, or that the sample size was insufficient to detect a significant difference (Fig. 2).

## Discussion

Our study estimated that the prevalence of cervicovaginal HPV infection in women aged 18 years or older was 16.4% in Ningbo, China. The most common HR-HPV genotypes were HPV 52, HPV 16, and HPV 58, whereas the prevalence of HPV 81, HPV 6, and HPV 11 was relatively low. The prevalence of vaginitis and cervicitis was significantly increased in HR-HPV-positive women. Pregnant women who were constantly infected with HR-HPV had a significantly increased risk of miscarriage and PROM. Our cross-sectional analysis revealed that the gestational glucose level was the most significant factor increasing the risk of HPV infection, while age and estrogen, and progesterone levels showed limited influence on the risk. Women with persistent HR-HPV infection had a significantly higher risk of reproductive tract inflammation and adverse pregnancy outcomes.

The HPV prevalence rate in our study was consistent with that in a previous national investigation in China (16.4% vs. 16.8%) [26], was slightly lower than that in a previous HPV epidemiology studies conducted in China (19%) [27]. The prevalence of HR-HPV infection varied widely among different regions in China, ranging from 12.2% in the northwestern region to 26.2% in the southwestern region [28, 29]. These regional differences in HPV prevalence can be attributed to the vast territory and unbalanced development in China, as well as the lack of economic resources and awareness regarding cervical cancer prevention and screening in certain underdeveloped areas. This can lead to nonstandard cervical cancer screening practices and result in geographic variations in HPV epidemiology. HPV 52 was the most common genotype in our study population, followed by HPV 16 and HPV 58. The higher prevalence of HPV 52 compared to HPV 16 in our study aligns with findings from other studies in China but were inconsistent with other studies in Western countries [30, 31, 32, 33]. The higher prevalence of HPV 52 compared to HPV 16 in our study population can be attributed to several factors that are supported by similar findings in other studies conducted in China. Here are some potential reasons and characteristics of these studies: [1] geographic variations: geographic differences in HPV genotype distribution are well-documented. Studies have shown that certain HPV types, including HPV 52, are more prevalent in East Asian populations. This could be due to genetic, environmental, and behavioral factors that influence the transmission and persistence of specific HPV types [2]. population characteristics: the demographic and clinical characteristics of the study population can impact the distribution of HPV genotypes. Factors such as age, sexual behavior, and immunological status can influence the prevalence of specific HPV types. For example, a younger population with a higher number of sexual partners may have a different HPV genotype distribution compared to an older population [3]. screening and detection methods: Variations in screening practices and the sensitivity of HPV detection methods can also contribute to differences in reported prevalence. Some studies might use more sensitive assays capable of detecting a wider range of HPV genotypes, leading to differences in prevalence rates [4]. cultural and behavioral factors: cultural practices and sexual behaviors, including the age of sexual debut and the number of sexual partners, can vary significantly between populations and influence HPV type distribution. HPV 16, HPV 45, HPV 31, HPV 33, and HPV 35 have been previously reported to be associated with a higher risk of cervical cancer than other oncogenic genotypes, but the oncogenicity of HPV genotypes may vary by race and population. Additionally, HPV 52 was categorized as a high-risk genotype by the International Agency for Research on Cancer, and its prevalence was extremely high in our study. Therefore, it’s emphasized that vaccination with HPV 9-valent vaccine is important among women of childbearing age in Ningbo, China.

As noted already, the prevalence of vaginitis and cervicitis was significantly higher in women with persistent HR-HPV infection, which was consistent with a previous study [20]. Previous studies have shown that an increased risk of various vaginitis and cervical infections in women with HPV infection [34]. Women infected with HPV suffer from a disrupted vaginal microbiota, leading to cervical mucosa dysbiosis and increased genital tract inflammation in sexually active individuals. It is still not entirely clear how concurrent HPV infection and genital tract inflammation affect the cervical epithelium, but growing evidence suggests that HPV-infected cervical epithelium is more susceptible to genital tract inflammation and the development of cervical cancer [35]. This represents the potential clinical importance of the combined detection of HR-HPV and lower genital tract infection in females.

Pregnancy is a risk factor for HPV infection in women due to changes in hormone levels and decreased immunity [36]. HR-HPV infection in women negatively impacts pregnancy and neonatal outcomes. Our study revealed that pregnant women who were HR-HPV-positive had significantly higher incidence rates of miscarriage and PROM, suggesting that HR-HPV-infected pregnant women are at increased risk for adverse pregnancy outcomes. This result is supported by previous studies that have shown that HR-HPV infection can lead to adverse pregnancy outcomes [37]. According to recent studies, HPV infection triggers the production of large amounts of prostaglandins, increasing the risk of adverse pregnancy outcomes, especially in the first trimester when the embryo is vulnerable to external factors [38]. Furthermore, HPV infection during pregnancy can also disrupt the balance of vaginal microbiota, leading to inflammation of the genital tract and an increased risk of adverse pregnancy outcomes [39]. Therefore, for women of reproductive age who have concurrent HPV infection and lower genital tract inflammation, it is recommended to consider pregnancy after treatment.

In our study, HR-HPV-positive pregnant women had significantly lower neonatal weights than HPV-negative pregnant women, while neonatal scores were not significantly different. Previous research has shown that women with HR-HPV infection face significant psychological barriers, most notably anxiety, guilt and self-blame, shame, and suspicion, all of which contribute to a decrease in their quality of life [40]. This may possibly explain the lower neonatal weights of newborns born to HR-HPV-positive pregnant women. At present, there are few studies on the impact of HPV infection on neonatal outcomes. It has been demonstrated that HPV may be transmitted through various channels, such as amniotic fluid, the placenta, blood, the birth canal, leading to fetal infection [41]. For neonatal HPV infection, the most serious consequence is juvenile-onset recurrent respiratory papillomatosis (JORRP), which is mainly caused by HPV 6 and HPV 11 infection and obstructs the respiratory tract of newborns, seriously affecting their quality of life, but its incidence rate is not high, at only 4.3 per 100 000 live births (51).

Age, smoking, the number of pregnancies, hormone changes, and pregnancy complications (such as gestational diabetes) are known to be associated with HPV infection during pregnancy [42]. Our results suggested that the prevalence of HPV infection decreased with age, which is consistent with some previous studies [16]. However, other studies have not shown a significant relationship between HPV infection and age [43]. We believe that glucose levels during pregnancy are associated with an increased risk of HPV infection. This finding was consistent with previous research, which claimed that diabetic patients are more likely to develop HPV infection during pregnancy, although the mechanism for this is not yet known [44]. Moreover, interaction is known to occur between HPV and some types of hormones, particularly estrogen and progesterone, resulting in persistent HPV infections. Women who expressed higher levels of estrogen and progesterone were significantly more likely to have cervical HPV infection. Nevertheless, this finding did not apply to pregnant women. Estradiol and progesterone levels in pregnant women were negatively associated with HPV infection in our study. This finding is likely a consequence of the protective effect of pregnancy hormones regulation on pregnant women.

## Conclusion

In conclusion, our study highlights that pregnant women with persistent high-risk HPV (HR-HPV) infections are at an increased risk of reproductive tract inflammation and adverse pregnancy outcomes. Specifically, HR-HPV infection is significantly associated with higher rates of miscarriage, lower live birth rates, increased cesarean section rates, and elevated risks of premature birth and low birth weight. Additionally, elevated glucose levels in pregnant women, particularly those with gestational diabetes mellitus, appear to be a notable factor associated with an increased risk of HPV infection. This underscores the importance of intensified monitoring of glucose levels in HPV-infected pregnant women to mitigate potential risks. However, it is important to note that bacterial vaginosis and other vaginal infections often co-exist with HPV infection. The directionality of this relationship-whether HPV infection increases the risk of these infections or vice versa-remains unclear. Therefore, while our findings suggest associations between HR-HPV infection and adverse pregnancy outcomes, conclusions about causality should be made with caution. Medical professionals should be aware of the increased risk of mother-to-child transmission of HR-HPV and the need for preventive measures, particularly for individuals with additional risk factors such as advanced age, gestational diabetes, and hormonal changes.

## Data Availability

The raw data supporting the conclusions of this article will be made available by the author Qi-fa Song, without undue reservation.

## Appendix

### Detailed obstetric examination procedures

Patients underwent prenatal and obstetric examinations including clinical cytological and microbiological examinations. Microbiological examinations, specifically including the vaginal microecology test, were conducted to assess the presence and balance of microorganisms in the vaginal environment. A physical examination of the genital area was performed to inspect the cervix and vagina for signs of inflammation such as redness, swelling, and discharge. A sample of cervical or vaginal discharge was taken for cytological examination to identify signs of cervicitis or vaginitis. Among pregnant women between 28 and 34 weeks of gestation, vaginal secretions were collected and individually placed in sterile tubes. The presence of chlamydia or gonorrhea was further tested using polymerase chain reaction (PCR) if necessary. PROM is generally diagnosed based on clinical manifestations and a physical examination. The physical examination for PROM usually requires a vaginal examination. During the vaginal examination, a large amount of amniotic fluid can be seen flowing out of the vagina for those who have a lot of fluid flow. For those with less fluid flow, it is generally necessary to use a speculum to stretch the vaginal wall to check whether there is amniotic fluid flowing out of the cervix and whether there is amniotic fluid pooling in the posterior fornix of the vagina.

### Diagnostic criteria for vaginitis

Vaginitis was diagnosed based on a combination of clinical symptoms and laboratory findings. Clinical symptoms included vaginal discharge, itching, irritation, and odor. Laboratory diagnosis involved microscopic examination of vaginal discharge samples to identify the presence of pathogenic organisms such as bacteria, fungi, and protozoa. Additionally, pH testing and the “whiff” test were performed to aid in the diagnosis.

### Diagnostic criteria for cervicitis

Cervicitis was diagnosed based on clinical examination and laboratory findings. Clinical examination included visual inspection of the cervix for signs of inflammation, redness, and discharge. Laboratory tests included cervical swabs for microbiological culture to identify causative pathogens and cytological examination to detect inflammatory cells.

### Data collection and classification

#### Timeline of data collection

HPV testing, laboratory tests, and clinical information were collected at various points during the study period (2016–2020). The timeline is as follows:

#### HPV testing

Conducted at the initial gynecological examination and repeated as needed based on clinical guidelines and patient history.

#### Laboratory tests and clinical information

Collected at multiple time points, including the initial examination, follow-up visits, and during pregnancy and delivery if applicable.

### Classification of information for analysis

#### HPV testing data

Used to determine HPV status and type. Women were classified based on their first positive HPV test result or their consistent negative status throughout the study.

#### Laboratory and clinical data

Analyzed based on the time of collection. For pregnant women, data were classified as either pre-pregnancy, during pregnancy, or at the time of delivery.

#### Pregnancy outcomes

Assessed based on clinical records and categorized as miscarriage, preterm birth, premature.
